# Pemphigus vulgaris in a neonate born to a mother with pemphigus vulgaris: A case report

**DOI:** 10.1002/ccr3.8343

**Published:** 2023-12-18

**Authors:** Ayush Anand, Praneet Awake, Anand Bhosale, Rajan Bindu, Anjali Kher, Prasad Bhanap

**Affiliations:** ^1^ BP Koirala Institute of Health Sciences Dharan Nepal; ^2^ Department of Dermatology, Venereology and Leprosy Symbiosis Medical College for Women, Symbiosis International (Deemed University) Pune Maharashtra India; ^3^ Department of Pathology Symbiosis Medical College for Women, Symbiosis International (Deemed University) Pune Maharashtra India; ^4^ Department of Paediatrics Symbiosis Medical College for Women, Symbiosis International (Deemed University) Pune Maharashtra India; ^5^ Department of Obstetrics and Gynaecology Symbiosis Medical College for Women, Symbiosis International (Deemed University) Pune Maharashtra India

**Keywords:** case report, neonate, pemphigus, pregnancy

## Abstract

**Key Clinical Message:**

Active pemphigus vulgaris in the mother can lead to neonatal pemphigus vulgaris, which is usually self‐limiting. Systemic corticosteroids are the mainstay of managing PV during pregnancy and until the child is breastfed.

**Abstract:**

Pemphigus vulgaris (PV) is a potentially life‐threatening autoimmune disease characterized by bullae and erosions over the skin and mucous membrane. PV is rarely reported in pregnant women and neonates. We reported the case of a 28‐year‐old Gravida 2 Parity 2 Living 1 who developed painful blisters and erosions in the oral cavity during third trimester of pregnancy. However, the diagnosis was delayed due to late presentation. The patient presented to our hospital at 37 weeks gestation with bullae and erosions distributed all over the body. Based on clinical evaluation and histopathology reports, she was diagnosed with PV. She delivered a child via cesarean section. The child also had similar lesions and was diagnosed with neonatal PV. Maternal PV was managed with prednisolone followed by azathioprine, leading to complete remission. No active intervention was required for neonatal PV as the condition was self‐limiting.

## Graphical abstract

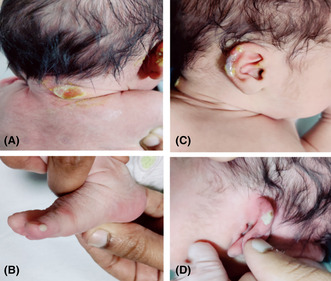

## 1 INTRODUCTION

Pemphigus vulgaris (PV) is a potentially fatal disease characterized by bullae and erosions over the skin and mucous membrane caused by autoantibodies to desmosomal adhesion proteins.^1^ PV is rarely reported in pregnant women and neonates and can sometimes lead to adverse maternal and fetal outcomes.^2^ Maternal PV can result in neonatal PV due to the passive transfer of IgG autoantibodies through the placenta.^1^ The evidence regarding the management option for PV in pregnancy and neonates is limited and requires an interdisciplinary team comprising obstetricians, dermatologists, and neonatologists.^3^, ^4^ Usually, immunosuppressants such as corticosteroids, intravenous immunoglobulin, mycophenolate mofetil, methotrexate, and azathioprine can treat PV.^3^, ^4^ However, in pregnant patients, mycophenolate mofetil, methotrexate, and azathioprine should be avoided.^5^ Nowadays, rituximab and corticosteroids are considered the first‐line agents for treating PV.^6^, ^7^, ^8^ Since neonatal PV is usually self‐limiting, it does not require specific intervention. In addition, the focus should be on promoting early healing and preventing associated infections by using emollients, topical corticosteroids, and antibiotics. Also, a one‐year follow‐up to assess the disease status is recommended.^9^

## 2 CASE REPORT

A 28‐year‐old Gravida 2 Parity 2 Living 1 at 37 weeks of gestation presented with erosions all over the body for 1 week associated with pruritus. Her past history revealed the occurrence of painful blisters and erosions in the oral cavity 7 months back. The patient took oral medications for the last episode, leading to the disappearance of lesions. Her personal and family history was unremarkable.

**FIGURE 1:**
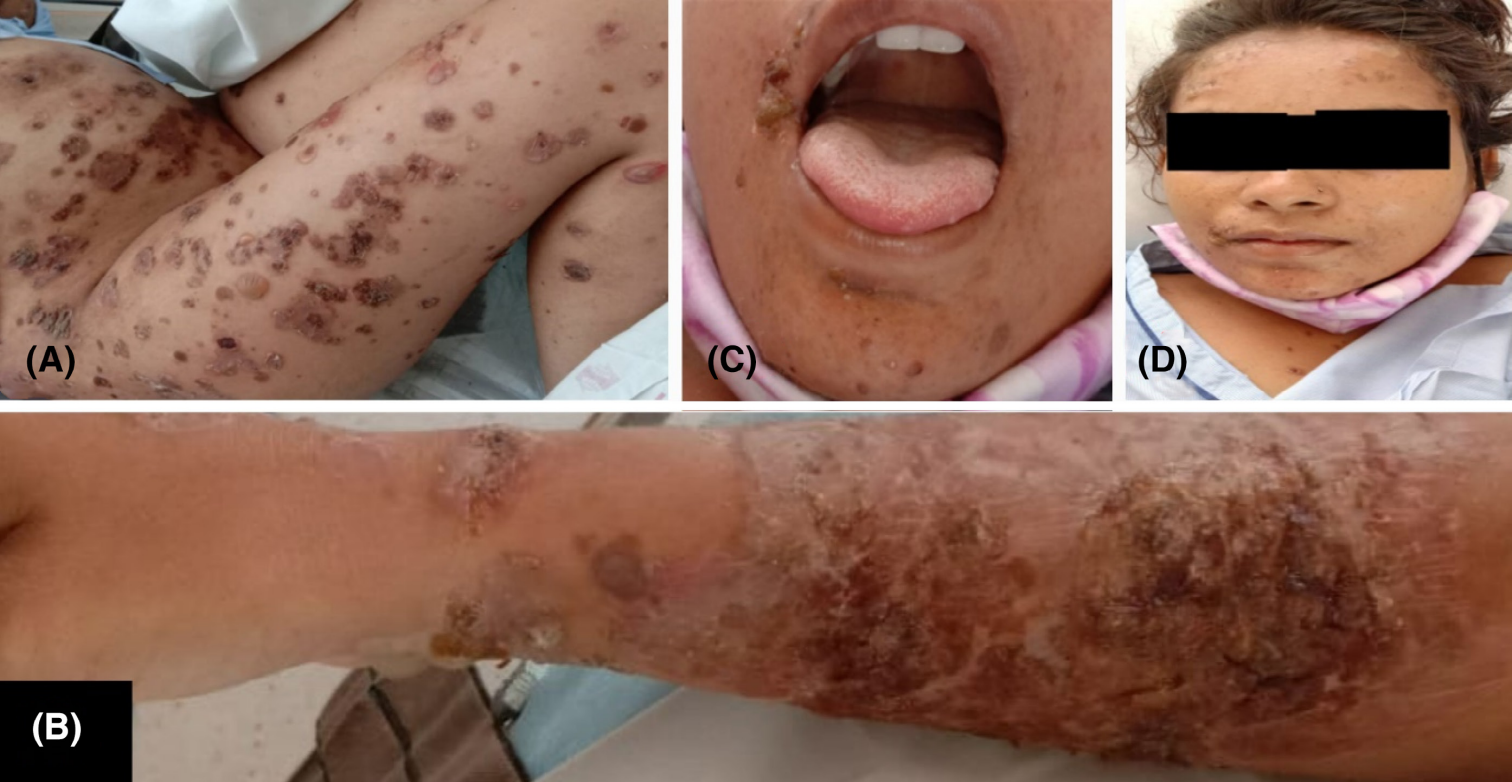
A. Multiple fluid‐filled flaccid bullae and erosions over the abdomen and lower limbs. B. Erosion with crusts on the right forearm. C. Perioral erosion with crusts on the face. D. Matting of hair with crusts on the face.

**FIGURE 2:**
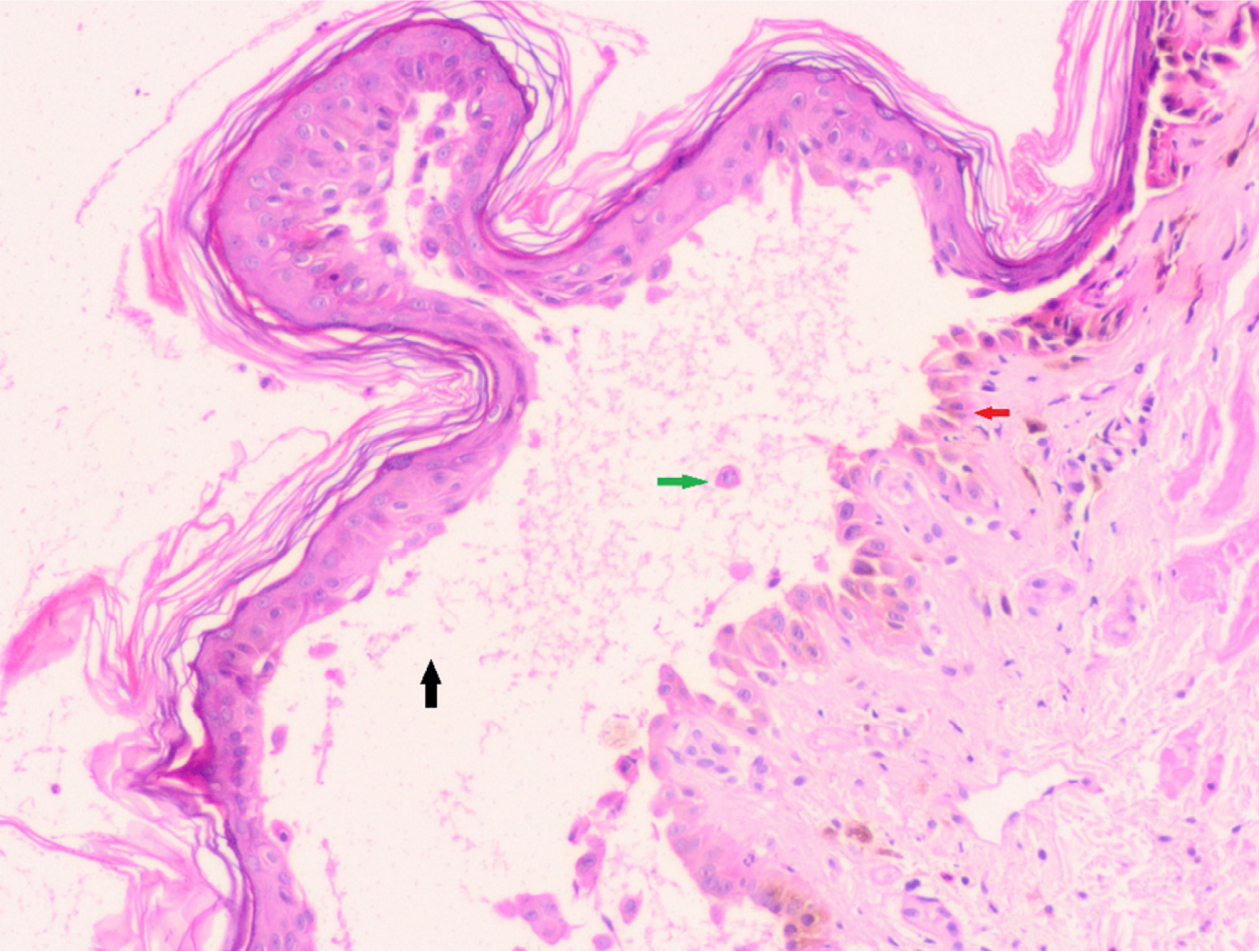
Skin biopsy of the mother showing the floor of the blister lined with mostly intact basal cells, giving a tombstone appearance (red arrow), suprabasal clefting (black arrow), and acantholytic cell (green arrow). Some cells seem to show spongiotic changes.

On examination, multiple fluid‐filled flaccid bullae and erosions were distributed all over the body, i.e., scalp (Figure 1D), face (Figure 1C), abdomen (Figure 1A), lower limbs (Figure 1A), upper limbs (Figure 1B). The oral cavity had few erosions. The genitals, palms, and soles were normal. Nikolsky's sign and Bulla's spread sign were positive. Initial lab investigations are mentioned in Table 1. To evaluate cutaneous lesions, we did a skin biopsy for an intact fresh blister over the arm of the mother (Figure 2). Histopathology revealed a suprabasal split in the epidermis, acantholytic cells, and basal cells arranged in a row like a tombstone. Some cells seem to show spongiotic changes. Direct immunofluorescence revealed IgG intercellular deposits in the epidermis in a fishnet pattern. Hence, we made a diagnosis of pemphigus vulgaris. However, desmoglein 1 and 3 autoantibodies testing was not done due to the unavailability of this test at our facility. Also, direct immunofluorescence testing was not done in the neonate.

**TABLE 1:** Laboratory investigations of the patient.

Investigations	Results	Reference range
Hb (g/dL)	12.2	13–16
RBC (million/mL)	4.68	1.5–4.5
TLC (cells/mm^3^)	14,300	4000–11,000
Platelets (cells/mm^3^)		
RBS (mg/dL)	100	70–150
Renal function test		
Serum sodium (mmol/L)	138	135–145
Serum potassium (mmol/L)	3.9	3.5–5.5
Serum urea (mg/dL)	7	15–40
Serum creatinine (mg/dL)	0.7	0.55–1.02
Liver function test		
Total protein (g/dL)	6.0	6.4–8.2
Albumin (g/dL)	1.7	3.4–1.5
ALT (IU/L)	14	18–65
AST (IU/L)	14	15–37
ALP (IU/L)	200	46–116
Thyroid function test		
T3	1.87	2.1–4.4
T4	0.8	0.8–2.7
TSH	1.53	0.4–4.2
Serology		
HIV	Negative	
HBsAg	Negative	
HCV	Negative	

Abbreviations: ALP, alkaline phosphatase; ALT, alanine transaminase; AST, aspartate transaminase; Hb, hemoglobin; HBsAg, Hepatitis B surface antigen; HCV, hepatitis C virus; HIV, human immunodeficiency virus; RBC, red blood cell; T3, triiodothyronine; T4, thyroxine; TLC, total leucocyte count; TSH, thyroid stimulating hormone.

**FIGURE 3:**
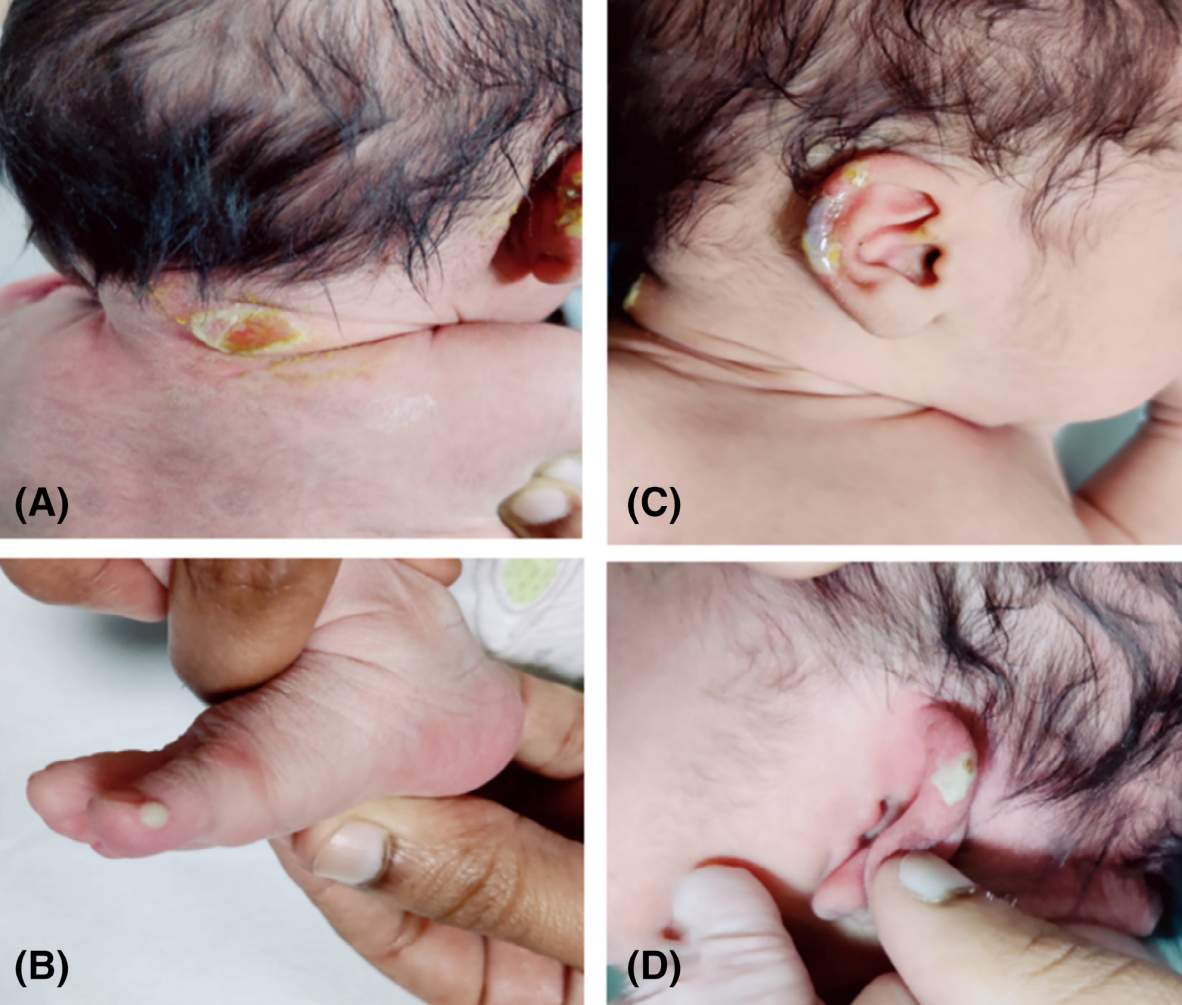
A. Blistering and vesicles on the neck. B. Blistering and vesicles on the foot of the neonate. C and D. Blistering and vesicles on the ear lobes.

**FIGURE 4:**
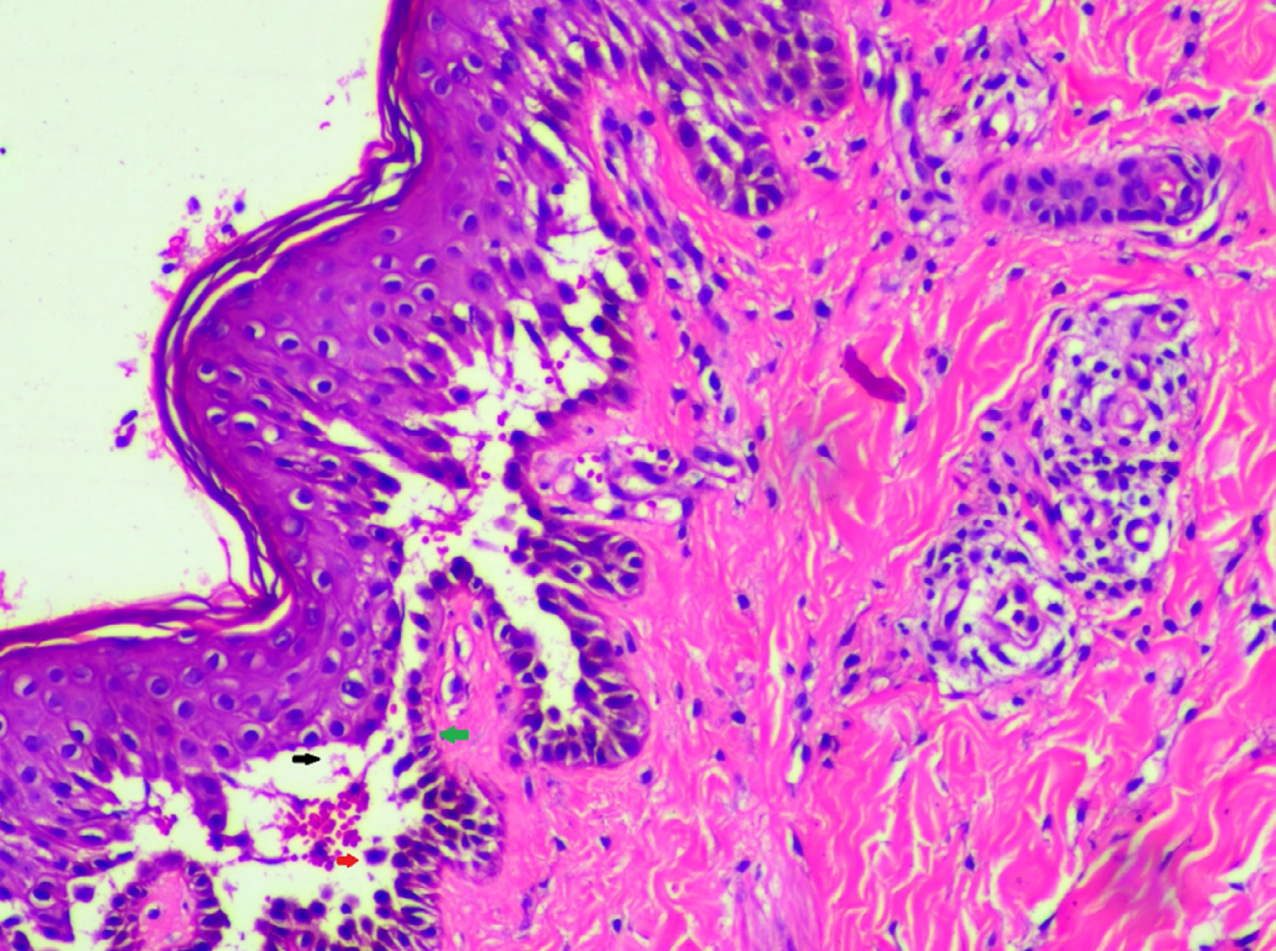
Skin biopsy of the neonate showing the floor of the blister lined with mostly intact basal cells, giving a tombstone appearance (red arrow), suprabasal clefting (black arrow), and acantholytic cell (green arrow). Some cells seem to show spongiotic changes.

We treated her with tablet prednisolone 40 mg once daily for 10 days. At 38 weeks gestation, the patient had thick meconium‐stained liquor and an unfavorable cervix. So, we managed the patient with emergency cesarean delivery. Furthermore, prednisolone was gradually tapered to 10 mg once daily over 6 months until the child was breastfed. In addition, local wound care was done to prevent infections. The neonate had blistering and vesicles on the neck (Figure 3A), ear lobes (Figure 3C and Figure 3D), and foot (Figure 3B). We did a skin biopsy of the fresh blister over the forearm in the infant, which suggested pemphigus vulgaris (Figure 4). As this condition is self‐limiting, we managed the neonate with topical mupirocin ointment. On follow‐up after 1 month, the infant was in remission, and lesions were healing in the mother. On subsequent follow‐ups, the mother had weight gain. So, prednisolone was stopped, and we kept her on tablet azathioprine 50 mg once daily. The mother and child are in remission on a recent follow‐up at 1 year.

## 3 DISCUSSION

Limited cases of pemphigus in pregnancy have been reported in the literature.^2^ The mean age of onset of maternal PV is 16 weeks of gestational period.^2^ Lin et al.^2^ reported that approximately 71% of the cases have PV, with most showing worsening or unchanged status. In most cases, both skin and mucosa are involved.^2^ Our patient developed bullae and erosions during the third month of gestation, and both skin and mucosa were involved in the mother. PV is characterized by a suprabasal split with a basal layer attached to the dermo‐epidermal basement membrane, giving a tombstone appearance.^10^ Direct immunofluorescence can reveal IgG deposits in the epidermis in a fish net pattern.^10^ The histopathology findings in both mother and child were consistent with PV. Based on clinical assessment and histopathology findings, we diagnosed PV in both mother and child.

Commonly used treatment approaches with azathioprine, mycophenolate mofetil, and methotrexate should be avoided in managing pemphigus in pregnant women.^5^, ^11^ These drugs can lead to premature birth and congenital defects in pregnancy.^5^ The studies have not suggested any deleterious effects of topical steroids and low‐dose systemic corticosteroids in pregnancy.^5^ Hence, systemic corticosteroids are the mainstay of managing PV during pregnancy and until the child is breastfed.^5^ Once the child stops breastfeeding, we can switch to alternatives such as azathioprine to avoid the long‐term side effects of corticosteroids.^4^, ^5^, ^10^ In our patient, we managed with low‐dosage prednisolone, gradually tapered to a minimal therapy dose. Later, due to weight gain in the mother, we switched to the alternative option of azathioprine. Usually, it takes approximately 3 to 8 months from diagnosis to complete remission.^12^ Majority of patients with PV report at least one relapse, with the median time from final diagnosis to relapse ranging between 18 and 44 months.^12^ We followed up with our patient for 1 year, and she did not report relapse. However, long‐term follow‐up is required in these patients to detect relapses. No specific treatment is necessary for neonatal PV, as the condition is self‐limiting in 2 to 3 weeks.^9^, ^13^ Hence, local wound care with topical emollients is sufficient. Similarly, we managed the neonate with local wound care and topical mupirocin ointment.

This case has certain limitations as desmoglein 1 and 3 autoantibodies testing was not done due to the unavailability of this test at our facility. Also, direct immunofluorescence testing was not done in the neonate.

## 4 CONCLUSION

Active pemphigus vulgaris in the mother can lead to neonatal pemphigus vulgaris. In pregnancy, the effective management of pemphigus vulgaris can be done using low‐dosage prednisolone with gradual tapering to a minimal dose in conjunction with local wound care. In addition, antibiotics should be given to prevent wound infection. Also, neonatal pemphigus vulgaris is self‐limiting, and no specific intervention is required.

## AUTHOR CONTRIBUTIONS

**Ayush Anand:** Conceptualization; supervision; validation; visualization; writing – original draft; writing – review and editing. **Praneet Awake:** Conceptualization; data curation; supervision; validation; visualization; writing – review and editing. **Anand Bhosale:** Data curation; validation; visualization; writing – review and editing. **Rajan Bindu:** Data curation; validation; visualization; writing – review and editing. **Anjali Kher:** Data curation; validation; visualization; writing – review and editing. **Prasad Bhanap:** Data curation; validation; visualization; writing – review and editing.

## FUNDING INFORMATION

The authors did not receive any funding for this paper.

## CONFLICT OF INTEREST STATEMENT

The authors have no conflicts of interest to declare.

## ETHICS STATEMENT

Our institution does not require ethical approval for reporting individual cases or case series.

## CONSENT

Written informed consent was obtained from the patient(s) for their anonymized information to be published in this article.

## GUARANTOR

PA is the guarantor for this manuscript.

## Data Availability

All relevant data pertaining to this case report is available within the manuscript.
